# Causal Relationships between Lymphocyte Subsets and Risk of Coronary Artery Disease: A Two-Sample Mendelian Randomization Study

**DOI:** 10.31083/j.rcm2509326

**Published:** 2024-09-11

**Authors:** Zhao Ma, Libo Liu, Jinfan Tian, Chenchen Tu, Dongfeng Zhang, Mingduo Zhang, Huan Zhang, Ziyu An, Meichen Sun, Hongjia Zhang, Xiantao Song

**Affiliations:** ^1^Department of Cardiology, Beijing Anzhen Hospital, Capital Medical University, 100029 Beijing, China; ^2^Department of Cardiovascular Surgery, Beijing Anzhen Hospital, Capital Medical University, 100029 Beijing, China

**Keywords:** lymphocyte subsets, coronary artery disease, myocardial infarction, Mendelian randomization

## Abstract

**Background:**

Currently, the causal relationship between lymphocyte subsets and coronary artery disease (CAD) remains unclear. Therefore, we utilized Mendelian randomization (MR) to assess the association between lymphocyte subsets and CAD.

**Methods:**

We performed a two-sample MR analysis using publicly available genome-wide association studies (GWAS) datasets. The primary method of analysis to comprehensively evaluate causal effects was the inverse variance-weighted (IVW) method. The four additional MR approaches were MR–Egger, weighted median, simple mode, and weighted mode. Sensitivity analysis incorporated Cochran's Q and MR–Egger intercept tests to identify residual heterogeneity and potential horizontal pleiotropy, respectively. The MR–PRESSO distortion test was applied to identify potential pleiotropic outliers. Leave-one-out analysis confirmed that no single single-nucleotide polymorphism (SNP) significantly affected the MR estimate. We conducted reverse MR analysis to investigate the impact of variables correlated with outcomes in forward MR analysis.

**Results:**

The IVW method revealed a significant positive association between B cell count and CAD (odds ratio (OR) = 1.08 (95% CI: 1.04, 1.11), *p* = 2.67 × 10^-5^). A similar association was observed between B cell count and myocardial infarction (MI) (OR = 1.07 (95% CI: 1.03, 1.11), *p* = 5.69 × 10^-4^). Sensitivity analyses detected no outliers, heterogeneity, or pleiotropy. The reverse MR analysis was conducted to investigate the impact of CAD and MI on B cell count, and the IVW results showed no statistical significance.

**Conclusions:**

Our study suggests that a higher absolute B cell count is linked to an increased risk of CAD and MI.

## 1. Introduction

Coronary artery disease (CAD), a foremost cardiovascular condition, has emerged as a principal global cause of mortality [1]. Myocardial infarction (MI), representing the most severe form of CAD, is marked by a significantly increased mortality rate. Atherosclerosis, the underlying pathophysiological mechanism of CAD, is recognized as an inflammatory disease [2]. Previous studies have illustrated the interplay between the innate and adaptive immune systems alongside cardiovascular risk factors, jointly contributing to the initiation and progression of atherosclerosis [3, 4].

Lymphocytes, which are integral to the immune response, include diverse cell types, such as CD4^+^ T cells, CD8^+^ T cells, regulatory T (Treg) cells, natural killer (NK) cells, and B cells. The array of lymphocyte subsets is a pivotal indicator of cellular and humoral immunity, reflecting the current immune function and homeostasis states. Previous research has indicated that the risk of CAD is heightened irrespective of variations in the total lymphocyte count [5, 6, 7]. Therefore, further exploration of the impact of specific lymphocyte subsets on CAD is imperative.

Numerous foundational experiments have highlighted the significant role of lymphocyte subsets in the development of atherosclerosis [3, 8, 9]. Despite these findings, analyzing lymphocyte subsets could be valuable for identifying biomarkers for the early diagnosis of CAD and pinpointing new therapeutic targets [10]. However, the analysis of lymphocyte subsets is not routinely practiced in clinical settings for CAD, and its clinical relevance for patients with CAD and MI is seldom reported. Furthermore, a recent cohort study contradicted earlier basic research by suggesting that the proportion of lymphocyte subsets in peripheral blood is not significantly associated with myocardial infarction or angina occurrence in adults without autoimmune diseases [11]. Therefore, clarifying the causal relationship between lymphocyte subset counts and CAD and MI is essential.

Mendelian randomization (MR) is an epidemiological technique used to infer potential causal relationships, which utilizes naturally occurring genetic variations as instruments [12]. These genetic variants are allocated randomly before birth and are established well before the onset of any disease. Consequently, MR helps reduce residual confounding and reverse causation problems often encountered in observational studies. Currently, no MR analysis has been conducted to examine the relationship between lymphocyte subsets and CAD and MI. Therefore, this MR analysis aimed to investigate the potential causal relationship between lymphocyte subsets and CAD and MI.

## 2. Materials and Methods

### 2.1 Study Design

**Fig. 1.**
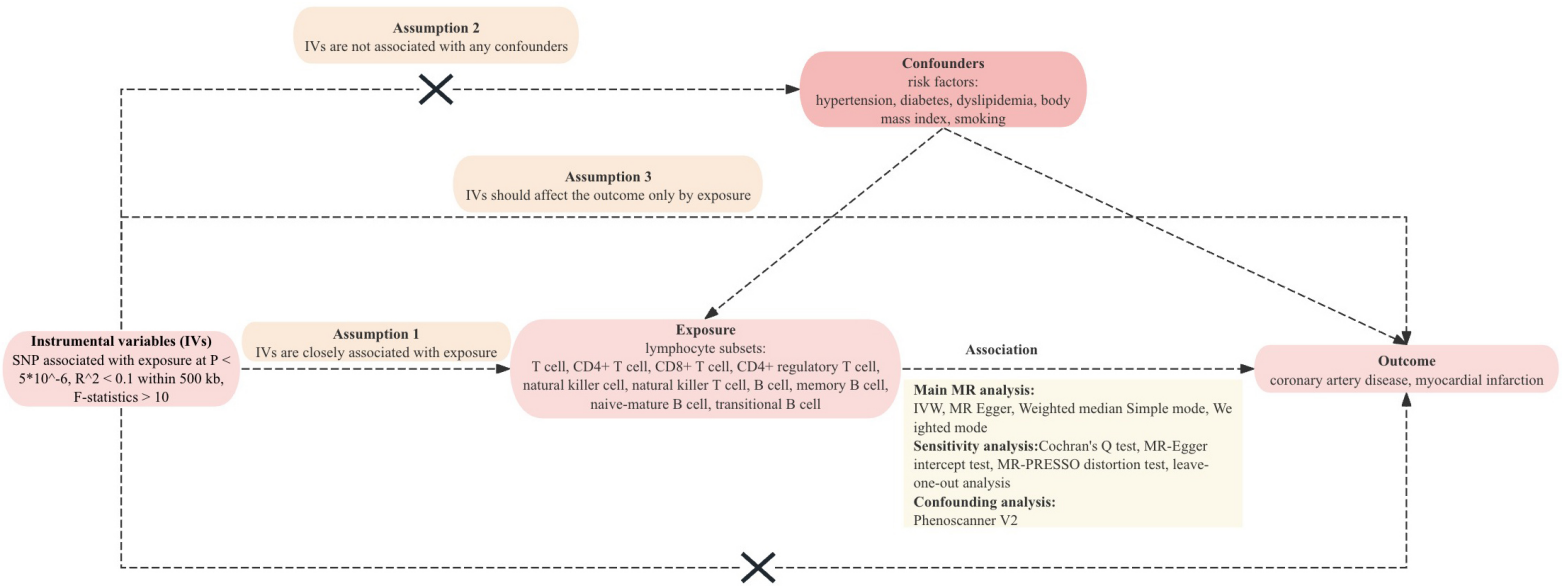
**Overview of overall MR design**. Abbreviations: IVs, instrumental variables; IVW, inverse variance-weighted; MR, Mendelian randomization; SNP, single-nucleotide polymorphism.

We conducted a two-sample MR study to explore the potential relationship between lymphocyte subsets and both CAD and MI. To ensure the validity of our findings, we selected instrumental variables (IVs) that adhered to three crucial criteria: (1) IVs must be strongly associated with the exposure; (2) IVs should not correlate with any confounders; (3) IVs should influence the outcome solely through exposure. Only IVs meeting all these conditions were included in our MR analysis [13]. As the data in this study are publicly accessible and detailed in the manuscript, obtaining ethical approval was unnecessary. Our overall study design is illustrated in Fig. 1.

### 2.2 Data Sources

The summary statistics for lymphocyte subsets from genome-wide association studies (GWAS), including T cells, CD4^+^ T cells, CD8^+^ T cells, CD4^+^ Tregs, NK cells, NKT cells, B cells, memory B cells, naive-mature B cells, and transitional B cells, were sourced from GWAS involving 3757 individuals from Sardinia [14]. The outcome data for CAD and MI were derived from the CARDIoGRAMplusC4D 1000 Genomes-based GWAS, which included data on 60,801 CAD cases and 123,504 controls [15]. The specific data sources utilized in this study are detailed in Table 1.

**Table 1.** **Data sources**.

Traits	GWAS ID or consortium	Sample size (cases/controls)	Ethnicity	Year
T cell	GCST90001603	Total: 3757	Sardinian	2020
CD4^+^ T cell	GCST90001590			
CD8^+^ T cell	GCST90001592			
CD4^+^ regulatory T cell	GCST90001513			
Natural killer cell	GCST90001648			
Natural killer T cell	GCST90001621			
B cell	GCST90001642			
Memory B cell	GCST90001407			
Naive-mature B cell	GCST90001409			
Transitional B cell	GCST90001577			
Coronary artery disease	CARDIoGRAMplusC4D 1000 Genomes-based GWAS	60,801/123,504	European and South Asian	2015
Myocardial infarction				

Table legend: GWAS, genome-wide association studies.

### 2.3 Instruments Selection

We adopted a stepwise method for selecting IVs. Initially, due to the limited number of single-nucleotide polymorphisms (SNPs) that reached genome-wide significance, we lowered the association threshold to *p* < 5 × 10^-6^ and eased the clumping threshold (R^2^ < 0.1 within a 500 kb distance), following precedents in previous research [16, 17]. To mitigate bias from using weak instruments, we calculated the F statistics for each SNP using the following formula: F = βeta^2^_exposure_/SE^2^_exposure_. Subsequently, we extracted SNPs from the outcome data, discarding those associated with the outcome (*p* < 1 × 10^-5^). Next, we harmonized the exposure and outcome SNPs to ensure allele alignment and eliminated palindromic SNPs with intermediate effect allele frequencies (effect allele frequency, EAF > 0.42) and SNPs with incompatible alleles. Furthermore, we utilized the Phenoscanner V2 website (http://www.phenoscanner.medschl.cam.ac.uk/) to exclude SNPs associated with outcome-related phenotypes (*p* < 1 × 10^-5^), including hypertension, diabetes, dyslipidemia, body mass index (BMI), and smoking.

### 2.4 Statistical Analysis

The random-effects inverse variance-weighted (IVW) method aggregates the Wald ratio of individual SNP to produce an aggregate estimate, assuming all genetic variants are valid. Thus, IVW is regarded as the most robust method for MR analysis [18]. We utilized IVW as the primary method to examine the association between exposure and outcome, complemented by four MR methodologies: MR–Egger, weighted median, simple mode, and weighted mode.

In secondary sensitivity analyses, Cochran’s Q test was used to evaluate residual heterogeneity (*p* < 0.05), while the MR–Egger intercept test was employed to detect potential horizontal pleiotropy (*p* < 0.05). Additionally, we identified potential pleiotropic outliers using the MR–PRESSO distortion test. A leave-one-out (LOO) analysis was also conducted to determine the impact of individual SNPs on the MR estimate. Considering the multiple tests performed in this study, we established a threshold for statistical significance at a *p*-value < 0.05/10. The causal association outcomes are presented as odds ratios (ORs) with 95% confidence interval (CI). These analyses were performed using the TwoSampleMR package (version 0.5.8) in R Studio (version 4.3.1, Posit Software, PBC, Boston, MA, United States).

### 2.5 Reverse Mendelian Randomization Analysis

In the reverse MR analysis, variables associated with CAD and MI identified in the forward MR analysis were selected for further examination. When considering CAD and MI as the exposures, we adjusted the association threshold to *p* < 5 × 10^-8^ and the clumping threshold (R^2^ < 0.001 within a 10,000 kb distance). Subsequent procedures were conducted following the guidelines outlined in the ‘Instrument Selection’ and ‘Statistical Analysis’ sections. Due to the multiple tests performed in this study, we established a threshold for statistical significance of a *p*-value < 0.05/2.

## 3. Results

### 3.1 Characteristics of the Selected SNPs

For CAD, following the selection of IVs, we employed 7 SNPs for T cells, 10 SNPs for CD4^+^ T cells, 19 SNPs for CD8^+^ T cells, 9 SNPs for CD4^+^ Treg cells, 43 SNPs for NK cells, 26 SNPs for NKT cells, 18 SNPs for B cells, 10 SNPs for memory B cells, 15 SNPs for naive-mature B cells, and 17 SNPs for transitional B cells. For MI, the selection of IVs led to the application of 7 SNPs for T cells, 10 SNPs for CD4^+^ T cells, 19 SNPs for CD8^+^ T cells, 9 SNPs for CD4^+^ Treg cells, 41 SNPs for NK cells, 25 SNPs for NKT cells, 18 SNPs for B cells, 11 SNPs for memory B cells, 15 SNPs for naive-mature B cells, and 17 SNPs for transitional B cells. All selected SNPs met our screening criteria (*p* < 5 × 10^-6^, R^2^ < 0.1, kb = 500). The F statistics for all were greater than 10, verified by the Phenoscanner V2 website. Details of the harmonized data are available in **Supplementary Table 1**.

### 3.2 Causal Relationship between Lymphocyte Subsets and CAD

We conducted MR analysis on the absolute counts of T cells, CD4^+^ T cells, CD8^+^ T cells, CD4^+^ Treg cells, NK cells, NKT cells, B cells, memory B cells, naive-mature B cells, and transitional B cells concerning CAD and MI. The findings indicated that higher absolute counts of B cells are associated with an increased risk of CAD (OR_IVW_ = 1.08 (1.04, 1.11), *p* = 2.67 × 10^-5^) (Fig. 2) and MI (OR_IVW_ = 1.07 (1.03, 1.11), *p* = 5.69 × 10^-4^) (Fig. 3). Other methods of analysis, including MR–Egger, weighted median, simple mode, and weighted mode, indicated a similar trend but without statistically significant differences (*p* > 0.05/10) (Figs. 4,5). However, evidence suggesting a causal relationship between other lymphocyte subsets and CAD or MI was not identified (*p* > 0.05/10). The detailed results of the MR analyses are depicted in Figs. 2,3. Scatter plots for the other lymphocyte subsets are provided in the **Supplementary Figs. 1–20**.

**Fig. 2.**
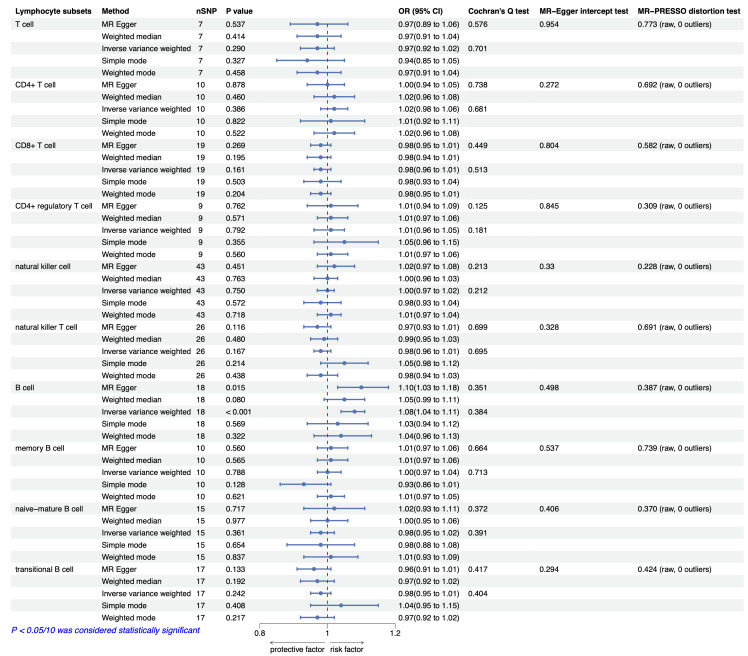
**MR analysis and sensitivity analysis results for the causal association between lymphocyte subsets and CAD**. Abbreviations: CAD, coronary artery disease; CI, confidence interval; MR, Mendelian randomization; OR, odds ratio; nSNP, the number of single-nucleotide polymorphism.

**Fig. 3.**
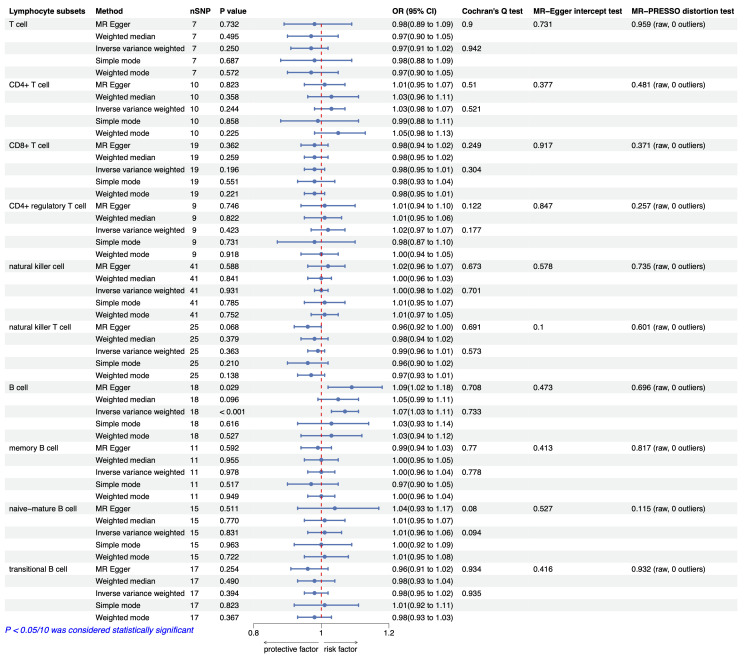
**MR analysis and sensitivity analysis results for the causal association between lymphocyte subsets and MI**. Abbreviations: CI, confidence interval; MI, myocardial infarction; MR, Mendelian randomization; OR, odds ratio; nSNP, the number of single-nucleotide polymorphism.

**Fig. 4.**
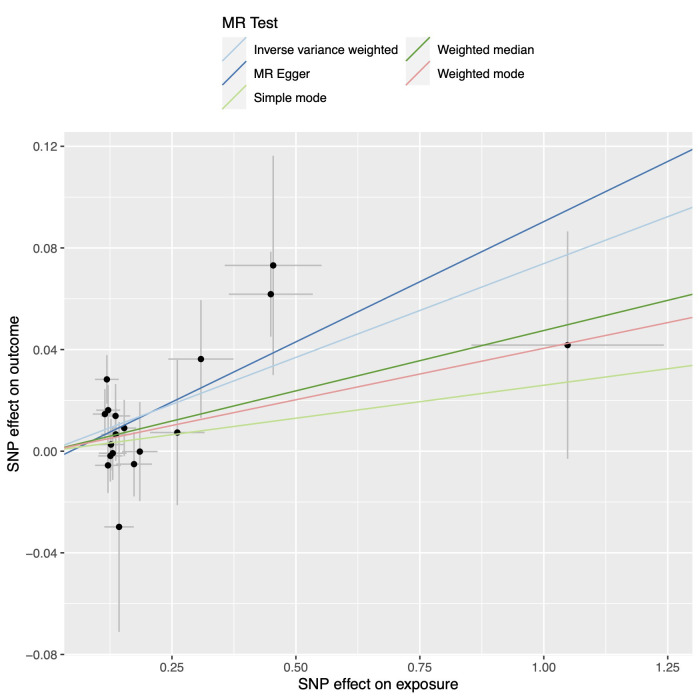
**Scatter plot of the association between B cells and CAD**. Abbreviations: CAD, coronary artery disease; MR, Mendelian randomization; SNP, single-nucleotide polymorphism.

**Fig. 5.**
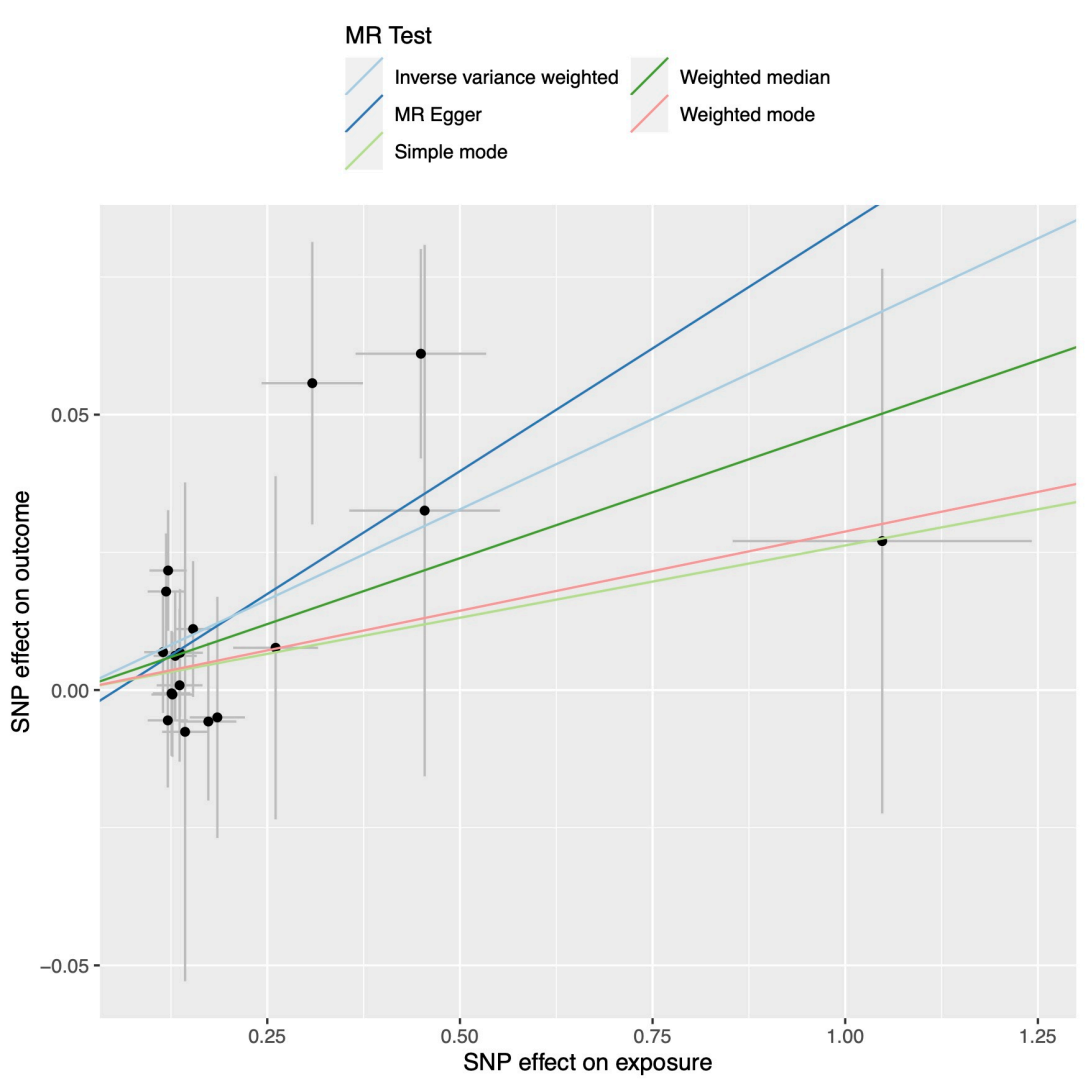
**Scatter plot of the association between B cells and MI**. Abbreviations: MR, Mendelian randomization; MI, myocardial infarction; SNP, single-nucleotide polymorphism.

### 3.3 Sensitivity Analysis

The MR–PRESSO analysis identified no outliers (*p* > 0.05). Cochran’s Q test revealed no heterogeneity in the results (*p* > 0.05), and the MR–Egger intercept test indicated no presence of horizontal pleiotropy (*p* > 0.05). Detailed results regarding heterogeneity and pleiotropy are displayed in Fig. 2. Furthermore, LOO sensitivity analysis revealed that the MR results remained unaffected by any individual SNP (see **Supplementary Figs. 19–38**).

### 3.4 Reverse Mendelian Randomization Analysis

In the forward MR analysis, only B cells exhibited a causal effect on CAD and MI. To investigate whether CAD and MI could influence B cell counts, reverse MR analyses were conducted. Following the selection of IVs, we utilized 36 SNPs for CAD and 22 SNPs for MI. All F statistics exceed the threshold of 10, verified using the Phenoscanner V2 website. The harmonized data are available in **Supplementary Table 1**.

The results indicated that CAD (OR_IVW_ = 0.91 (0.82, 1.01), *p* = 0.0741) and MI (OR_IVW_ = 0.87 (0.76, 0.99), *p* = 0.0312) did not significantly affect B cell counts (*p* > 0.05/2). Additionally, the MR–PRESSO analysis revealed no outliers, Cochran’s Q test showed no heterogeneity (*p* > 0.05), and the MR–Egger intercept test detected no significant pleiotropy (*p* > 0.05). The MR findings and scatter plots are presented in **Supplementary Table 2** and **Supplementary Figs. 39,40**. Details on heterogeneity and pleiotropy are also included in **Supplementary Table 2**. LOO sensitivity analysis is detailed in **Supplementary Figs. 41,42** and confirmed the reliability of the reverse MR results.

## 4. Discussion

In this study, we conducted a two-sample MR analysis to investigate the causal relationship between lymphocyte subsets and both CAD and MI for the first time. The results demonstrated a significant association between increased B cell counts and a higher risk of CAD and MI. These findings remained consistent after adjusting for potential confounders and applying the Bonferroni correction. Furthermore, the causal relationship was supported by sensitivity analyses, while a reverse MR analysis was performed to exclude the possibility of reverse causation between B cell counts and these outcomes.

Atherosclerosis, the primary pathological process underlying CAD, involves the accumulation of lipids and immune cells within the arterial walls, forming atherosclerotic plaques [2]. Substantial research using preclinical models, along with emerging clinical evidence, has underscored the pivotal roles of both the innate and adaptive immune systems in promoting inflammation associated with atherosclerosis in arterial blood vessels [19]. Study analyzing atherosclerotic plaques in mice and humans have identified T cells and macrophages as the predominant immune cells present [20]. However, some studies have also found B cell-enriched tertiary lymphoid organs within atherosclerotic lesions [21, 22, 23]. Additionally, circulating immunoglobulins, which originate from secondary lymphoid organs and are found within atherosclerotic lesions in both human and animal models, suggest a possible role for B cells outside the lesions in the pathogenesis of the disease [24, 25, 26].

B cells play a crucial role in innate and adaptive immunity by participating in antigen presentation, cytokine secretion, and the production of autoantibodies. Additionally, mouse-derived B cells have been extensively studied owing to their accessibility for research. These B cells are typically categorized into B1 and B2 based on their distinct characteristics and cell surface markers. B1 cells, originating from the fetal liver, primarily reside in the serosal cavities and have the capacity for self-renewal in the periphery [27]. They can migrate to the spleen and bone marrow, where they produce IgM antibodies [27]. Mouse-derived B1 cells are further classified into B1a (CD5+) and B1b (CD5-) cells based on the presence and absence of CD5 molecules, respectively [28]. B2 cells, arising from lymphoid progenitors, mature in the bone marrow and undergo further maturation in secondary lymphoid organs. They differentiate into follicular B cells and marginal zone B cells before entering the peripheral circulation [29].

The mechanisms underlying the involvement of B cells in atherosclerosis have been thoroughly investigated in mice, with distinct functions identified for the B1 and B2 subgroups [9, 30, 31]. B1 cells, especially B1a cells, demonstrate anti-atherosclerotic properties by secreting natural IgM antibodies. These antibodies target oxidized low-density lipoprotein (ox-LDL), reducing necrotic cores and apoptosis within lesions [24]. Notably, B1a cells can secrete IgM without antigen activation, whereas both antigenic and non-antigenic stimuli can activate B1b cells. This activation produces antigen-specific IgM and small quantities of IgG, with B1b cells eventually transitioning into memory B cells [32]. Contrastingly, B2 cells, the predominant B cell type in peripheral circulation, contribute to atherosclerosis development by producing pathogenic IgG antibodies, activating T cells, and promoting the release of proinflammatory cytokines. Specifically, mouse-derived B2 cells produce IgG that induces inflammation [33]; they also play a role in atherosclerosis development by secreting TNF-α, increasing macrophage tumor necrosis factor-α (TNF-α) production, and promoting cell death and inflammation [34]. Our study identified a correlation between the counts of peripheral circulating B cells and CAD. Despite these differences between species, this finding aligns with the proatherogenic role attributed to B2 cells, which constitute the majority of B cells in the peripheral circulation.

However, the findings from studies on mouse-derived B cells cannot be directly extrapolated to humans [35]. Additionally, there has been limited research and varying conclusions concerning the relationship between human-derived B cells and CAD [36]. Therefore, we conducted MR analyses using available databases to bridge this knowledge gap. In humans, B cells are categorized into transitional B cells, naive B cells, memory B cells, and plasmablast subtypes, distinguished by their expression levels of CD19, CD20, CD24, CD38, CD27, and IgD production [37]. Our MR analyses specifically targeted memory B cells, naive-mature B cells, and transitional B cells from the database. However, the results did not reveal any association between the counts of these B cell subtypes and CAD. This lack of association could be due to the limited sample sizes available in the GWAS database.

We further analyzed MR to investigate the association between lymphocyte subset subtypes and MI. Our MR analysis revealed a positive correlation between the circulating B cell count and the risk of MI. However, further analysis focusing on B lymphocyte subtypes did not produce significant results. Kyaw *et al*. [38] reported that MI could accelerate the progression of atherosclerosis and that the depletion of B cells may prevent the accumulation of IgG antibodies in atherosclerotic plaques, thus attenuating the MI-induced acceleration of atherosclerosis. During MI, alarm molecules such as heat shock protein-60, high mobility group box-1, and mitochondrial DNA are released, triggering the activation of T cells and B cells [39, 40]. The activated B cells further stimulate monocyte mobilization and enhance antibody production in response to antigens released during MI, ultimately resulting in myocardial damage [41]. Since activated B cells can differentiate into long-lived plasma cells, the pathogenic antibodies produced during MI can persist for years, continuously contributing to the progression of atherosclerosis [42].

Our analysis found no significant association between any lymphocyte subsets, apart from B cells, and CAD and MI. This absence of association might be due to the possibility that changes in the circulating numbers of other lymphocyte subsets do not directly influence the development of atherosclerosis. For instance, CD8^+^ T cells could aggravate atherosclerosis by activating follicular B cells within secondary and tertiary lymphoid organs [42]. Moreover, the interaction between B cells and CD4^+^ T cells may augment their pro-atherosclerotic effects, even though the circulating CD4^+^ T cell count does not change in atherosclerotic mice [43].

In clinical practice, the analysis of lymphocyte subsets has been employed to evaluate the severity and stage of diseases. It is essential to acknowledge that factors such as age, sex, race, and smoking habits can affect the composition of lymphocyte subsets in healthy individuals [44]. Therefore, in selecting the exposure and outcome databases for our study, we meticulously ensured consistency across the sample populations and excluded SNPs associated with hypertension, diabetes, dyslipidemia, BMI, and smoking.

Current targeted therapies against B cells have yet to be widely implemented in atherosclerosis treatment. However, both prior research findings and our analysis support the theoretical viability of this approach. Various potential therapeutic strategies focusing on B cells for atherosclerosis have been explored in animal models. For example, immunization with antigens such as pneumococci, malondialdehyde-modified low-density lipoprotein (LDL), and Apolipoprotein B100 (ApoB100) has been shown to stimulate B cells to produce oxLDL-specific IgM, which may help reduce atherosclerosis [45, 46]. Additionally, in mice treated with CD20-specific monoclonal antibodies, a reduction in B2 cells and a decrease in IgG secretion were observed, leading to a diet-induced decrease in atherosclerosis [47]. Stimulating B cells to produce antibodies that protect against atherosclerosis through vaccination or similar methods appears to be a promising strategy. Nevertheless, our conclusion contradicts the findings of a nested case–control study conducted by Olson *et al*. [11], which highlighted potential difficulties in utilizing circulating B cell count as a biomarker for CAD. Thus, additional research is needed to investigate and validate this approach thoroughly.

Our study has several limitations. First, we relied on a single GWAS database for lymphocyte subsets with a relatively small sample size, which may affect the robustness of our conclusions. Second, due to the modest scale of GWAS data available for lymphocyte subsets, we reduced the association threshold to *p* < 5 × 10^-6^ and relaxed the clumping threshold (R^2^ < 0.1 within a 500 kb distance). Third, the CAD database we utilized only categorizes myocardial infarction and does not differentiate among other types of CAD, necessitating further MR analyses to separate CAD into more specific diagnostic categories.

## 5. Conclusions

In this study, we conducted a two-sample partial two-way Mendelian randomization analysis using a publicly available GWAS database. We focused on 10 lymphocyte subsets to explore their potential association with the risk of CAD. Our findings suggest that higher B cell counts are associated with an increased risk of CAD and MI. Therefore, B cell counts in peripheral blood may be a potential predictive marker for CAD and MI.

## Availability of Data and Materials

All data points generated or analyzed during this study are included in this article and there are no further underlying data necessary to reproduce the results.

## Abbreviations

CI, confidence interval; CAD, coronary artery disease; GWAS, genome-wide association studies; IVs, instrumental variables; IVW, inverse variance-weighted; LOO, leave-one-out; LDL, low-density lipoprotein; MI, myocardial infarction; MR, Mendelian randomization; NK, natural killer; OR, odds ratio; ox-LDL, oxidized low-density lipoprotein; Treg, regulatory T; SNP, single-nucleotide polymorphism.

## Supplementary Material

Supplementary material associated with this article can be found, in the online version, at https://doi.org/10.31083/j.rcm2509326.
