# Supplementary material for: Causal Relationships between Lymphocyte Subsets and Risk of Coronary Artery Disease: A Two-Sample Mendelian Randomization Study
Source: Rev Cardiovasc Med. 2024 Sep 11;25(9):326. doi: 10.31083/j.rcm2509326 (PMC11440411; doi:10.31083/j.rcm2509326)
Supplement: Supplementary file 1 [file 2153-8174-25-9-326-s1.zip › Supplementary material-Figs.docx]

Supplementary Fig. 1. Scatter plot of association between T cell and CAD

Figure legend: CAD, coronary artery disease; MR, mendelian randomization; SNP, single-nucleotide polymorphism

Supplementary Fig. 2. Scatter plot of association between T cell and MI

Figure legend: MI, myocardial infarction; MR, mendelian randomization; SNP, single-nucleotide polymorphism

Supplementary Fig. 3. Scatter plot of association between CD4+T cell and CAD

Figure legend: CAD, coronary artery disease; MR, mendelian randomization; SNP, single-nucleotide polymorphism

Supplementary Fig. 4. Scatter plot of association between CD4+T cell and MI

Figure legend: MI, myocardial infarction; MR, mendelian randomization; SNP, single-nucleotide polymorphism

Supplementary Fig. 5. Scatter plot of association between CD8+T cell and CAD

Figure legend: CAD, coronary artery disease; MR, mendelian randomization; SNP, single-nucleotide polymorphism

Supplementary Fig. 6. Scatter plot of association between CD8+T cell and CAD

Figure legend: MI, myocardial infarction; MR, mendelian randomization; SNP, single-nucleotide polymorphism

Supplementary Fig. 7. Scatter plot of association between CD4+ Treg cell and CAD

Figure legend: CAD, coronary artery disease; MR, mendelian randomization; SNP, single-nucleotide polymorphism

Supplementary Fig. 8. Scatter plot of association between CD4+ Treg cell and MI

Figure legend: MI, myocardial infarction; MR, mendelian randomization; SNP, single-nucleotide polymorphism

Supplementary Fig. 9. Scatter plot of association between natural killer cell and CAD

Figure legend: CAD, coronary artery disease; MR, mendelian randomization; SNP, single-nucleotide polymorphism

Supplementary Fig. 10. Scatter plot of association between natural killer cell and MI

Figure legend: MI, myocardial infarction; MR, mendelian randomization; SNP, single-nucleotide polymorphism

Supplementary Fig. 11. Scatter plot of association between natural killer T cell and CAD

Figure legend: CAD, coronary artery disease; MR, mendelian randomization; SNP, single-nucleotide polymorphism

Supplementary Fig. 12. Scatter plot of association between natural killer T cell and MI

Figure legend: MI, myocardial infarction; MR, mendelian randomization; SNP, single-nucleotide polymorphism

Supplementary Fig. 13. Scatter plot of association between memory B cell and CAD

Figure legend: CAD, coronary artery disease; MR, mendelian randomization; SNP, single-nucleotide polymorphism

Supplementary Fig. 14. Scatter plot of association between memory B cell and MI

Figure legend: MI, myocardial infarction; MR, mendelian randomization; SNP, single-nucleotide polymorphism

Supplementary Fig. 15. Scatter plot of association between naive-mature B cell and CAD

Figure legend: CAD, coronary artery disease; MR, mendelian randomization; SNP, single-nucleotide polymorphism

Supplementary Fig. 16. Scatter plot of association between naive-mature B cell and MI

Figure legend: MI, myocardial infarction; MR, mendelian randomization; SNP, single-nucleotide polymorphism

Supplementary Fig. 17. Scatter plot of association between transitional B cell and CAD

Figure legend: CAD, coronary artery disease; MR, mendelian randomization; SNP, single-nucleotide polymorphism

Supplementary Fig. 18. Scatter plot of association between transitional B cell and MI

Figure legend: MI, myocardial infarction; MR, mendelian randomization; SNP, single-nucleotide polymorphism

Supplementary Fig. 19. The result of leave-one-out sensitivity analysis of T cell and CAD

Figure legend: CAD, coronary artery disease; MR, mendelian randomization

Supplementary Fig. 20. The result of leave-one-out sensitivity analysis of T cell and MI

Figure legend: MI, myocardial infarction; MR, mendelian randomization

Supplementary Fig. 21. The result of leave-one-out sensitivity analysis of CD4+ T cell and CAD

Figure legend: CAD, coronary artery disease; MR, mendelian randomization

Supplementary Fig. 22. The result of leave-one-out sensitivity analysis of CD4+ T cell and MI

Figure legend: MI, myocardial infarction; MR, mendelian randomization

Supplementary Fig. 23. The result of leave-one-out sensitivity analysis of CD8+ T cell and CAD

Figure legend: CAD, coronary artery disease; MR, mendelian randomization

Supplementary Fig. 24. The result of leave-one-out sensitivity analysis of CD8+ T cell and MI

Figure legend: MI, myocardial infarction; MR, mendelian randomization

Supplementary Fig. 25. The result of leave-one-out sensitivity analysis of CD4+ regulatory T cell and CAD

Figure legend: CAD, coronary artery disease; MR, mendelian randomization

Supplementary Fig. 26. The result of leave-one-out sensitivity analysis of CD4+ regulatory T cell and MI

Figure legend: MI, myocardial infarction; MR, mendelian randomization

Supplementary Fig. 27. The result of leave-one-out sensitivity analysis of natural killer cell and CAD

Figure legend: CAD, coronary artery disease; MR, mendelian randomization

Supplementary Fig. 28. The result of leave-one-out sensitivity analysis of natural killer cell and MI

Figure legend: MI, myocardial infarction; MR, mendelian randomization

Supplementary Fig. 29. The result of leave-one-out sensitivity analysis of natural killer T cell and CAD

Figure legend: CAD, coronary artery disease; MR, mendelian randomization

Supplementary Fig. 30. The result of leave-one-out sensitivity analysis of natural killer T cell and MI

Figure legend: MI, myocardial infarction; MR, mendelian randomization

Supplementary Fig. 31. The result of leave-one-out sensitivity analysis of B cell and CAD

Figure legend: CAD, coronary artery disease; MR, mendelian randomization

Supplementary Fig. 32. The result of leave-one-out sensitivity analysis of B cell and MI

Figure legend: MI, myocardial infarction; MR, mendelian randomization

Supplementary Fig. 33. The result of leave-one-out sensitivity analysis of memory B cell and CAD

Figure legend: CAD, coronary artery disease; MR, mendelian randomization

Supplementary Fig. 34. The result of leave-one-out sensitivity analysis of memory B cell and MI

Figure legend: MI, myocardial infarction; MR, mendelian randomization

Supplementary Fig. 35. The result of leave-one-out sensitivity analysis of naive-mature B cell and CAD

Figure legend: CAD, coronary artery disease; MR, mendelian randomization

Supplementary Fig. 36. The result of leave-one-out sensitivity analysis of naive-mature B cell and MI

Figure legend: MI, myocardial infarction; MR, mendelian randomization

Supplementary Fig. 37. The result of leave-one-out sensitivity analysis of transitional B cell and CAD

Figure legend: CAD, coronary artery disease; MR, mendelian randomization

Supplementary Fig. 38. The result of leave-one-out sensitivity analysis of transitional B cell and MI

Figure legend: MI, myocardial infarction; MR, mendelian randomization

Supplementary Fig. 39. Scatter plot of association between CAD and B cell

Figure legend: CAD, coronary artery disease; MR, mendelian randomization; SNP, single-nucleotide polymorphism

Supplementary Fig. 40. Scatter plot of association between MI and B cell

Figure legend: MI, myocardial infarction; MR, mendelian randomization; SNP, single-nucleotide polymorphism

Supplementary Fig. 41. The result of leave-one-out sensitivity analysis of CAD and B cell

Figure legend: CAD, coronary artery disease; MR, mendelian randomization

Supplementary Fig. 42. The result of leave-one-out sensitivity analysis of MI and B cell

Figure legend: MI, myocardial infarction; MR, mendelian randomization
