# Supplementary material for: Causal Relationships between Lymphocyte Subsets and Risk of Coronary Artery Disease: A Two-Sample Mendelian Randomization Study
Source: Rev Cardiovasc Med. 2024 Sep 11;25(9):326. doi: 10.31083/j.rcm2509326 (PMC11440411; doi:10.31083/j.rcm2509326)
Supplement: Supplementary file 1 [file 2153-8174-25-9-326-s1.zip › Supplementary material-Table 2.docx]

Supplementary Table 2. The result of reverse MR analysis and sensitivity analysis for causal association between CAD/MI and B cell

| Exposure | Method | nSNP | P value | OR (95% CI) | Cochrane’s Q test | MR-Egger intercept test | MR-PRESSO distortion test |
| --- | --- | --- | --- | --- | --- | --- | --- |
| CAD | MR egger | 36 | 0.282 | 0.87 (0.69, 1.11) | 0.718 | 0.728 | 0.783 (raw, 0 outliers) |
|  | Weighted median |  | 0.223 | 0.91 (0.78, 1.06) |  |  |  |
|  | inverse variance weighted |  | 0.0741 | 0.91 (0.82, 1.01) | 0.753 |  |  |
|  | Simple mode |  | 0.285 | 0.85 (0.64, 1.14) |  |  |  |
|  | weighted mode |  | 0.237 | 0.89 (0.75, 1.07) |  |  |  |
| MI | MR egger | 22 | 0.514 | 0.9 (0.66, 1.23) | 0.28 | 0.788 | 0.364 (raw, 0 outliers) |
|  | Weighted median |  | 0.113 | 0.86 (0.72, 1.03) |  |  |  |
|  | inverse variance weighted |  | 0.0312 | 0.87 (0.76, 0.99) | 0.33 |  |  |
|  | Simple mode |  | 0.404 | 0.86 (0.62, 1.21) |  |  |  |
|  | weighted mode |  | 0.278 | 0.89 (0.72, 1.09) |  |  |  |

Legend: CAD, coronary artery disease; MI, myocardial infarction; MR, mendelian randomization; SNP, single-nucleotide polymorphism; OR, odds ratios; CI, confidence intervals
